# Antagonistic Mechanism of Iturin A and Plipastatin A from *Bacillus amyloliquefaciens* S76-3 from Wheat Spikes against *Fusarium graminearum*

**DOI:** 10.1371/journal.pone.0116871

**Published:** 2015-02-17

**Authors:** An-Dong Gong, He-Ping Li, Qing-Song Yuan, Xiu-Shi Song, Wei Yao, Wei-Jie He, Jing-Bo Zhang, Yu-Cai Liao

**Affiliations:** 1 Molecular Biotechnology Laboratory of Triticeae Crops, Huazhong Agricultural University, Wuhan 430070, People’s Republic of China; 2 College of Plant Science and Technology, Huazhong Agricultural University, Wuhan 430070, People’s Republic of China; 3 College of Life Science and Technology, Huazhong Agricultural University, Wuhan 430070, People’s Republic of China; Korea University, KOREA, REPUBLIC OF

## Abstract

Controlling toxigenic *Fusarium graminearum* (FG) is challenging. A bacterial strain (S76-3, identified as *Bacillus amyloliquefaciens*) that was isolated from diseased wheat spikes in the field displayed strong antifungal activity against FG. Reverse-phase high performance liquid chromatography and electrospray ionization mass spectrometry analyses revealed that S76-3 produced three classes of cyclic lipopeptides including iturin, plipastatin and surfactin. Each class consisted of several different molecules. The iturin and plipastatin fractions strongly inhibited FG; the surfactin fractions did not. The most abundant compound that had antagonistic activity from the iturin fraction was iturin A (*m/z* 1043.35); the most abundant active compound from the plipastatin fraction was plipastatin A (*m/z* 1463.90). These compounds were analyzed with collision-induced dissociation mass spectrometry. The two purified compounds displayed strong fungicidal activity, completely killing conidial spores at the minimal inhibitory concentration range of 50 µg/ml (iturin A) and 100 µg/ml (plipastatin A). Optical and fluorescence microscopy analyses revealed severe morphological changes in conidia and substantial distortions in FG hyphae treated with iturin A or plipastatin A. Iturin A caused leakage and/or inactivation of FG cellular contents and plipastatin A caused vacuolation. Time-lapse imaging of dynamic antagonistic processes illustrated that iturin A caused distortion and conglobation along hyphae and inhibited branch formation and growth, while plipastatin A caused conglobation in young hyphae and branch tips. Transmission electron microscopy analyses demonstrated that the cell walls of conidia and hyphae of iturin A and plipastatin A treated FG had large gaps and that their plasma membranes were severely damaged and separated from cell walls.

## Introduction

*Fusarium graminearum* (FG) Schwabe (teleomorph *Gibberella zeae* (Schwein) Petch) is a globally important plant pathogen that is responsible for the devastating diseases Fusarium head blight (FHB) of wheat and *Gibberella* ear rot of maize [1–3]. Between 1998 and 2000, economic losses attributed to FHB in the US were estimated at 3 billion dollars [4]. FHB epidemics in wheat occur frequently in central China, especially along the middle and lower reaches of the Yangtze River [5–7]. Recently, such epidemics have spread to an even wider area in China. Beyond FG-induced yield losses, *Fusarium* pathogens produce various types of trichothecene mycotoxins in grains that are highly toxic to humans and domestic animals [8].

During the past 30 years, crop protection strategies against FHB have relied heavily on the application of chemical fungicides, a practice that has resulted in undesirable environmental and ecological consequences [9]. Incidence of fungicide-resistant *Fusarium* pathogens in wheat fields has increased dramatically in many regions of China since the mid-1990s [10]. Therefore, alternatives to the current chemical control methods must be found to reduce yield losses of crops and to lower mycotoxin loads in food/feed chains. Biocontrol agents that are friendly to the environment and ecosystems have recently attracted more and more attention all over the world. *Bacillus* spp., with proven colonization aptitude and outstanding sporulation ability have been well studied and are frequent candidates for use as biocontrol agents [11].

*Bacillus* spp. are effective as biocontrol agents against plant pathogens due primarily to their production of various cyclic lipopeptides [12]. These cyclic lipopeptides are of the iturin (such as bacillomycin D/F/L/Lc, iturin A/C, and mycosubtilin), fengycin (fengycin A/B, and plipastatin A/B), and surfactin (halobacillin, pumilacidin and surfactin) classes, which all share a common structure consisting of a lipid tail linked to a short cyclic peptide. Among the lipopeptides, iturin and fengycin have been shown to have antifungal activity [13], whereas, surfactin showed no marked fungitoxicity [16]. Studies have shown that iturin forms ion-conducting pores that increase the electrical conductance of artificial lipid membranes [14]. Iturin is also known to disturb the cytoplasmic membranes of yeast cells, causing leakage of K^+^ ions and other vital constituents in parallel with the death of yeast cells [15,16]. Mycosubtilin (the most active form in iturin family) produced by *B*. *subtilis* was strongly active against different yeast species but inactive against *Aspergillus* spp. [17]. Subsequent studies with mycosubtilin and artificial membranes showed that the activity of the lipopeptides was dependent on the interactions with phospholipid and sterols, especially with the acyl chains of the phospholipid [18] and alcohol group of cholesterol in the membranes [19]; mycosubtilin displayed a preferential affinity to cholesterol (the main sterol in animal membranes) over ergosterol (the main fungal sterol). A recent study showed that iturin can be used for biological control of FG in field test and that lower iturin levels on wheat spikes could be a major factor limiting disease control [20].

Fengycin has been shown to have a variety of bioactivities: it is known to inhibit fungal growth [21] and to cause perturbation, bending, and micelle formation on artificial membranes [22–24]. Fengycin has no obvious effects on the morphology or cell structure of *Fusarium oxysporum* [25] and no effect on yeast [21]. Fengycin often caused pore formation of the membranes through all-or-none mechanism; low concentrations of fengycin showed no effect on the membrane, whereas at high enough concentrations it caused large sustainable pores, allowing for the complete efflux of intercellular contents of affected cells [26]. The composition of phospholipids and sterols in membranes was considered to be related with the antifungal activity of fengycin [26]. As for the nomenclature of fengycin, a recent study has structurally clarified that the plipastatin identified by Umezawa et al. in 1986 and the fengycin reported by Budzikiewicz et al. in 1999 are actually identical compounds that display slightly structural variations at different salty conditions [27]. Thus, the term plipastatin is used throughout this study.

Various reports have shown that lipopeptides produced by *Bacillus* spp. showed antagonistic activity against several agronomically- and medicinally-important fungal pathogens including *Botrytis cinerea* [28], *Candida albicans* [29], *Fusarium graminearum* [30], and *Podosphaera fusca* [13]. These lipopeptides are also active against the bacterial pathogens *Vibrio anguillarum* and *Shewanella aquimarina* [31]. Different *Bacillus* spp. produced varied types of active lipopeptides. For instance, the coproduction of chitinase, fengycin and surfactin was considered to contribute to inhibition of *F*. *graminearum* by *B*. *subtilis* strain SG6 [30]. Previous reports have mainly focused on the antifungal activity of total lipopeptide extracts. No study to date has examined and compared the individual contributions of particular lipopeptides to the antagonistic mechanism of such lipopeptides from *Bacillus* species against the same filamentous fungal pathogen. Further no report has characterized the dynamic antagonistic processes of these lipopeptides as they act to alter fungal growth and development.

To evaluate the antagonistic action of individual lipopeptides from one *Bacillus* sp. against FG, we initially isolated one antagonist *Bacillus amyloliquefaciens* strain (S76–3) from diseased wheat spikes in the field. Using reverse-phase high performance liquid chromatography (RP-HPLC) and electrospray ionization mass spectrometry (ESI-MS), we identified three classes of lipopeptides from this bacterial strain, including iturin, plipastatin and surfactin. Iturin and plipastatin showed antifungal activity. Iturin A and plipastatin A were the most abundant molecules of their respective lipopeptide classes, and both had activity against FG. Their structures were characterized by collision-induced dissociation mass spectrometry (CID-MS). The two different compounds showed distinct antagonistic effects against FG. Microscopic and time-lapse imaging analyses revealed that iturin A caused substantial condensation and conglobation along hyphae and severe restriction of branch formation, while plipastatin A mainly caused vacuolation and conglobation on young hyphae and branch tips. Transmission electron microscopy (TEM) showed that treatment of FG with either compound caused widely gapped cell walls and disturbed plasma membranes. The results of these separate image analyses indicated that iturin A and plipastatin A have different antagonistic mechanisms against FG, but that both compounds gave rise to the same deleterious cellular consequences in FG. The isolated *Bacillus* strain S76–3 that can produce large quantities of both compounds has significant potential for use as a biocontrol agent for controlling *Fusarium* pathogens in agricultural production systems.

## Materials and Methods

### Ethics statement

Specific permission was not needed for our field samples. The strains used in our study were isolated from natural environment. *B*. *amyloliquefaciens* strain S76–3 was isolated for spikes of wheat grown in our own experiment fields in our university, Wuhan, China (geographical coordinate at N: 30°28′11.34″, E: 114°21′29.86″). *F*. *graminearum* 5035 was isolated from *Fusarium*-infected wheat spikes also collected from our own experimental fields, Wuhan, as indicated in citation. The field studies did not involve endangered or protected species. No transgenic or created mutant microbes have been used, and no vertebrate studies were performed in our study.

### Microbe strains and culture conditions

Healthy or diseased wheat spikes showing light pink or orange coloration (symptomatic of Fusarium head blight) were collected from experimental fields in Wuhan. The samples were surface sterilized with 75% ethanol (v/v) for 30 s and 0.1% (w/v) HgCl_2_ for 30 s. After drying on sterile filter paper, the samples were cut into pieces about 3–4 mm in length, placed on potato dextrose agar (PDA) plates, and incubated at 28°C to culture microorganisms. Single bacterial colonies with different characteristics were re-streaked on NA (beef extract 3 g/L, peptone 10 g/L, NaCl 5 g/L, agar 15 g/L, pH 7.2) plates to obtain pure cultures. All isolates were stored in 25% glycerol (v/v) at -70°C for further use.

A single colony of *Bacillus* strain S76–3 (collection number M2014315 at the China Center for Type Culture Collection) that was isolated from weakly diseased wheat spikes (wheat cultivar Annong 8455) was inoculated in YPG medium (yeast extract 10 g/L, tryptone 20 g/L, dextrose 20 g/L, pH 7.2) for 48 h to produce cyclic lipopeptides. FG 5035 (collection number AF 2014011 at CCTCC) from a scabby wheat spike in Wuhan, China is a deoxynivalenol-producer and a highly pathogenic strain [6]; it was used throughout this study. FG 5035 was grown at 28°C on PDA plates for fresh mycelium propagation, and grown in CMC medium for conidiation [32]. Minimal inhibitory concentration (MIC) and microscopy analyses of iturin A and plipastatin A against FG conidia and hyphae were conducted in half-strength YPG medium.

### Screening and identification of selected antagonists

A dual cultural test was used to screen for effective antagonists against FG 5035. Briefly, a fresh 5-mm diameter hyphal disk was placed on the center of a PDA plate, and a single colony of the selected bacterium was inoculated 3 cm from the fungal disk. The plates were sealed with plastic membranes and cultured at 28°C in the dark. The inhibition rate of each strain against FG mycelium growth was tested 5 days post inoculation (dpi). The antagonists with dramatic antifungal activity were selected for subsequent molecular, physiological, and biochemical analyses. To classify the selected strains, bacterial genomic DNA was extracted using a previously described method [33]. 16S rDNA sequences were amplified by polymerase chain reaction (PCR) with two primers, 27f (5’ to 3’: AGAGTTTGATCCTGGCTC) and 1541R (5’ to 3’: AAGGAGGTGATCCAGCCGCA) [34]. Amplified segments were sequenced by BGI (BGI, Shenzhen, China), and evaluated with BLAST analysis (http://www.ncbi.nlm.nih.gov/blast). A phylogenetic tree of 16S rDNA sequences was constructed using MEGA 3.1 [31]; the tree contained the 16S rDNA sequences of strain S76–3 and other 13 closely-related *Bacillus* species downloaded from the NCBI database. Physiological and biochemical analyses were conducted according to previously described methods [35,36]. These analyses included carbon utilization, gelatin liquefaction, milk peptonization, salt and high temperature tolerance, oxidase reactions, and starch and casein hydrolysis.

### Production and extraction of lipopeptides

A single S76–3 colony was inoculated into 20 ml of YPG medium in a 100 ml flask and cultured at 28°C for 18 h. 12 ml of this culture was then inoculated into 200 ml of YPG medium in a 500 ml flask and cultured for 48 h. The supernatant was collected following centrifugation at 12,000 x g for 20 min at 4°C. Lipopeptides in the supernatant were precipitated and collected as previously described [37], with the following modifications: precipitates were washed 2 times with distilled water, redissolved in methanol, and adjusted to pH 7.0 with 1.0 N NaOH. The samples were loaded onto a HiCapt C18 solid phase extraction (SPE) column (500 mg, 6 ml), washed with 24 ml acetonitrile/water (10/90, v/v, HPLC grade), and eluted with 24 ml acetonitrile/water (80/20, v/v). After evaporation *in vacuo*, the products were dissolved in 1 ml of methanol and analyzed by RP-HPLC and ESI-CID-MS.

### RP-HPLC and mass spectrometry analyses of lipopeptides

Lipopeptides extracted from strain S76–3 were analyzed with a RP-HPLC (Agilent Technologies Co., Palo Alto, CA, USA) system equipped with an Agilent C18 column (150 × 4.6 mm, 5 µm particle diameter) and a UV detector. Mobile phase A was acetonitrile with 0.1% (v/v) trifluoroacetic acid (TFA) (Sigma-Aldrich, St. Louis, MO, USA), and mobile phase B was Milli-Q water with 0.1% (v/v) TFA. The lipopeptides were eluted at a flow rate of 1 ml/min with a linear gradient of solvent A, developed from 10% to 100% over 60 min. The elution pattern was monitored by determining absorbance at 214 nm [25,37,43]. Authentic reference standards for iturin A and surfactin (Sigma-Aldrich, St Louis, MO) were used to construct standard curves for the quantitative analysis of lipopeptides produced by strain S76–3. Fractions were collected, concentrated *in vacuo*, and further analyzed with ESI-CID-MS. As no commercial plipastatin is available, plipastatin A purified from RP-HPLC was identified by ESI-CID-MS, weighted and used for the construction of standard curves for quantitative analyses in this study.

Mass spectrometry analyses were performed using an LC-MS-8030 system (Shimadzu, Kyoto, Japan) with a triple quadruple mass analyzer and an ESI source, in positive full scan mode. The interface voltage was 4.5 kv (ESI+); the detector voltage was 1.2 kv; the desolvation gas temperature was 250°C; the heat block temperature was 400°C. Nitrogen was used as the nebulizer gas at a flow rate of 3.0 L/min and dry gas flow at 15 L/min. CID was performed using argon as the collision gas at a pressure of 230 kpa, and the collision energy was optimized for each precursor ion selected: -40 ev for iturin and -70 ev for plipastatin.

### Biocidal activity of purified lipopeptides against FG

Iturin, plipastatin, and surfactin fractions were collected with an RP-HPLC system and subsequently evaluated for antifungal activity against FG. The three fractions were collected following separation with a semi-preparative XDB-C18 column (9.4 × 250 mm, 5 µm particle diameter, Agilent Technologies, USA) at a flow rate of 4 ml/min, with the method described above. Following evaporation *in vacuo*, the extracts were weighed on an AB 135-S single pan electronic balance (Mettler Toledo, Schwerzenbach, Switzerland, d = 0.01 mg) and then redissolved in methanol to a final concentration of 10 mg/ml. Aliquots of 20 µl of each fraction were inoculated onto filter disks that were placed 3 cm away from an FG plug on PDA plates. 20 µl of methanol was used as a control. The biocidal activity of each compound was evaluated 4 dpi.

Iturin A and plipastatin A were collected and used to determine their MIC against FG conidia. For MIC tests, iturin A and/or plipastatin A were serially diluted, in two-fold steps, from 500 µg/ml to 10 µg/ml in half-strength YPG medium to a final volume of 250 µl in 2 ml plastic tubes; the same volume of fresh FG conidia was added to each tube to a final concentration of 1 × 10^5^ spores/ml. Reactions omitting antagonistic compounds were used as controls. All reactions were cultured at 200 rpm at 28°C for 12 h. Germinated conidia were recorded under a microscope by randomly counting 40 conidia in each replicate. Germ tubes that were equal to or longer than the length of conidia were considered to have germinated. The experiments were conducted for three times, with three replicates in each time. All MIC data were combined to calculate the rate of inhibition of conidia germination and subjected to analysis of variance (ANOVA) using SAS 9.2 software (SAS Institute, Cary, NC, USA). Differences among the individual tests and controls were determined by using Duncan’s multiple range tests (P < 0.05). The germination inhibition rate was calculated according to the following formula: Inhibition rate (%) = [(the control germination rate—the treated germination rate)/ the control germination rate] × 100 [25]. The MIC refers to the concentration at which more than 95% conidia did not germinate in 12 h. To check whether the conidia treated with iturin A or plipastatin A really lost the capacity to germinate, conidia treated at MIC for 12 h were collected by centrifugation (12,000 × g, 4°C, 10 min), resuspended in 500 µl H_2_O, and then plated on PDA plates and cultured at 28°C for three days.

### Optical, epifluorescence, and transmission electron microscopy

Optical and epifluorescence microscopy was carried out using a Nikon eclipse 90i microscope (Nikon, Tokyo, Japan) with GFP-HQ and UV fluorescence filters and Plan Apo objectives of 20 × (0.75 NA) and 40 × (0.95 NA). Normanski images were captured using a DS-Fi1 camera (Nikon) and epifluorescence images were captured using an Andor Clara camera (Andor, Belfast, Northern Ireland). For fluorescence analysis, the filters were set at an excitation wavelength of 455–485 nm and an emission wavelength of 495 nm. Elements software v3.22 (Nikon) was used for microscope control and image analysis [38].

Conidial spores (1 × 10^5^ spores/ml) or young hyphae germinated for 6 h from conidia (1 × 10^5^ spores/ml) were used for the microscopy analyses. Conidia and young hyphae treated with iturin A (50 µg/ml) or plipastatin A (100 µg/ml) in half-strength YPG medium for 12 h were prepared for microscopy after staining with 50 μg/ml fluorescein diacetete (FDA) (Sigma-Aldrich, St. Lousi, Mo, USA) for 8 min in the dark. Images were taken randomly from three independent experiments. The inhibition and dynamic antagonistic processes of iturin A and plipastatin A against FG were monitored by capturing time-lapse images at 5 frames per second at room temperature for a period of 200 min. Prior to the time-lapse analysis, young hyphae were cultured with iturin A (50 µg/ml) or plipastatin A (100 µg/ml) in half strength YPG medium for 6 h. Videos of changes in the hyphae were then captured under a microscope for a period of 200 min at room temperature, with the cultural medium added to each glass slide, respectively.

TEM was used to evaluate the structural characteristics of conidia and young hyphae treated with iturin A (50 µg/ml) or plipastatin A (100 µg/ml) for 12 h in half-strength YPG medium. The spores and hyphae were fixed in 2.5% (v/v) glutaraldehyde and 1% (v/v) osmium tetroxide. Sections were prepared and visualized using an H-7650 transmission electron microscope (Hitachi, Tokyo, Japan), as described by Xu et al. [32].

## Results

### Isolation of antagonists against *Fusarium graminearum* from wheat spikes

**Fig 1:**
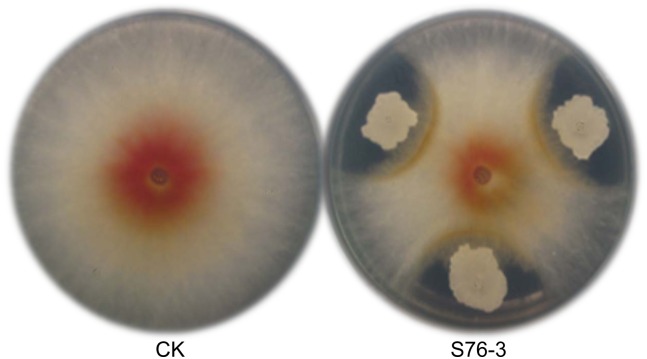
Antifungal activity of *B*. *amyloliquefaciens* S76–3 against FG on PDA plates 5 dpi (28°C). Fungal plug was placed in the center of PDA plate and S76–3 was inoculated 3 cm from the fungal plug (S76–3). Fungal plug inoculated on PDA plate center was used as control (CK).

To isolate effective antagonists against FG, a total of 175 microbes isolated from healthy (123 strains) and scabby (52 strains) wheat spikes were assayed for their antifungal activities by dual cultural methods *in vitro*. Strain S76–3, isolated from a weakly scabby wheat spike in Wuhan (wheat cv. Annong 8455), showed strong antagonistic activity (Fig. 1) against FG growth and development, in both growth chamber and field plot assays (data not show). This strain was screened as a potential biocontrol agent and subsequently identified by molecular and physiological analyses. The 16S rDNA sequence of S76–3 (GenBank ID in NCBI: JQ267647) had high similarity to those of *B*. *subtilis* and *B*. *amyloliquefaciens* (S1 Fig.). Moreover, S76–3 showed lactose utilization, brown color on potato blocks, tolerance to NaCl at 1.71 M, and good growth at 51°C in S1 Table, indicating that strain S76–3 was *B*. *amyloliquefaciens* [35,36,39].

### HPLC and ESI mass spectrometry analysis of lipopeptides produced by S76–3

**Fig 2:**
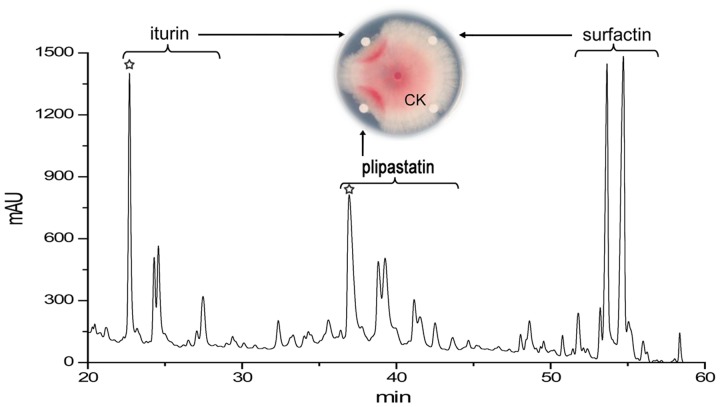
RP-HPLC analysis of lipopeptides from strain S76–3, and detection of their antagonistic activity against FG. Iturin, plipastatin, and surfactin were analyzed and collected by RP-HPLC, and their antifungal activities were detected on PDA plates 4 dpi (28°C). Aliquots of 20 µl of each (10 mg/ml) were added to filter disks and placed 3 cm away from the fungal plug. 20 µl of methanol on a filter disk was used as control.

RP-HPLC analysis revealed three main classes of compounds (Fig. 2), two of which had the same retention times (iturin, 22.755–27.586 min; surfactin, 51.704–54.893 min) and UV spectra (214 nm) as did two reference lipopeptides (a mix of iturin and surfactin, respectively) purchased from Sigma in S2 Fig. Further ESI-MS analyses showed that the three classes of compounds had similar molecular weights with the three types of cyclic lipopeptides produced by *Bacillus* species: iturin, plipastatin, and surfactin [16,40]. There were molecular ion peaks (M+H)^+^ for iturin at *m/z* 1043 and 1057, for plipastatin at *m/z* 1436, 1450, 1464, 1478, 1492, and 1506, and for surfactin at *m/z* 1008, 1022, 1036, and 1050. All of these molecules within each class had a 14 Da difference in molecular weights, suggesting the presence of varied lengths of fatty acid chains within each group (CH_2_ = 14 Da). Structural elucidation of iturin and plipastatin with ESI-CID-MS was described below.

Assays testing the three classes of compounds revealed that iturin and plipastatin strongly inhibited mycelium growth of FG, but that surfactin did not show discernible activity against FG (Fig. 2, inset small figure). Next, further fractionation of the two active classes yielded two molecules from iturin and five molecules from plipastatin that were antagonistically active against FG. The fractions from iturin and plipastatin were used for subsequent ESI-CID-MS analysis.

### ESI-CID-MS spectrometry analysis of iturin A and plipastatin A

**Fig 3:**
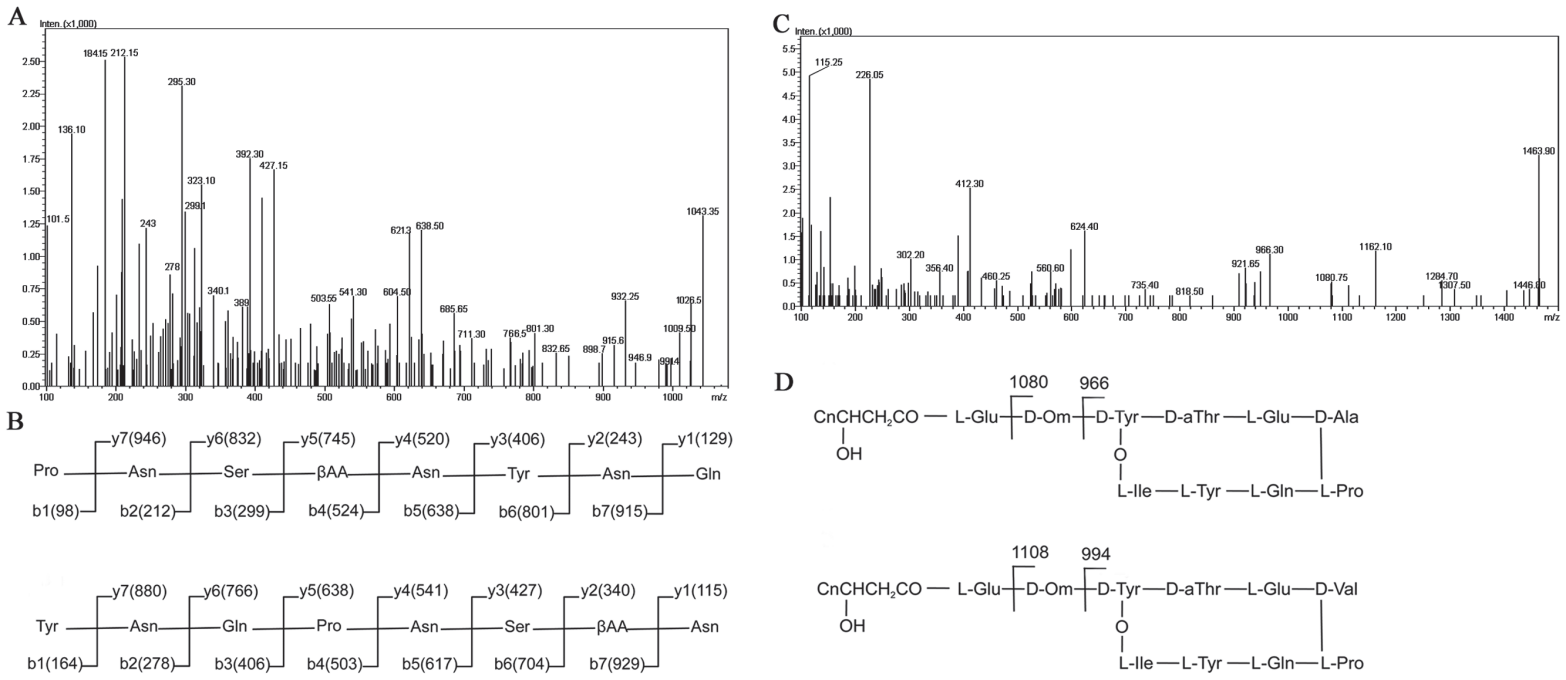
ESI-CID-MS analysis of iturin A (*m/z* 1043.35) and plipastatin A (*m/z* 1463.90). CID spectrum of precursor ion of iturin at *m/z* 1043.35 (A), and the theoretical b-type and y-type fragment ions dissociated from ring-opening reactions at Gln-Pro (B, top panel) and Asn-Tyr (B, bottom panel). CID spectrum of precursor ion of plipastatin A at *m/z* 1463.90 (C), and common breakage and two typical fingerprint ions for identification of plipastatin A (D, top panel) and plipastatin B (D, bottom panel) molecules. Carbon number in acyl acid chains for iturin A (*m/z* 1043.35) is 14 (βAA), and for plipastatin A (*m/z* 1463.90) is 16 (n = 13).

ESI-CID-MS analysis was performed using iturin A (*m/z* 1043.35) and plipastatin A (*m/z* 1463.90) as precursor ions (Fig. 2, two major fractions marked with asterisks). As shown in Fig. 3A, from the CID spectrum of iturin precursor ion, an ion at *m/z* 184.15 was detected and identified to be the immonium ion of the β-amino acid (C_11_H_23_-CH = N^+^H_2_), which was the long fatty acid chain cleaved from the cyclic peptide. In the identification of cyclic peptides, the peptide ring should be opened at a characteristic peptide bond to form a linear acylium ion; the other peptide bonds can then be broken to generate a range of fragment ions that can be detected in the CID spectrum. The presence of a proline in cyclic lipopeptides promotes breakage at Xaa-Pro bonds that generates a Pro-Asn-Ser-βAA-Asn-Tyr-Asn-Gln-Co^+^ fragment (P-N-S-X-N-Y-N-Q-CO^+^, βAA and X denotes β—amino acid) (Fig. 3B, top panel) [41]. The b-type fragment ions at *m/z* 212 (b2), *m/z* 299 (b3), *m/z* 524 (b4), *m/z* 638 (b5), *m/z* 801 (b6), and *m/z* 915 (b7), and the y-type fragment ions at *m/z* 243 (y2), *m/z* 406 (y3), *m/z* 520 (y4), and *m/z* 946 (y7) were also detected in the CID spectrum (Fig. 3A).

Another major linear acylium ion with ring opening at Xaa-Tyr was also detected in the CID spectrum of the iturin precursor, generating the linear amino acid sequence Tyr-Asn-Gln-Pro-Asn-Ser-βAA-Asn-CO^+^ (Y-N-Q-P-N-S-X-N-CO^+^) (Fig. 3B, bottom panel) [41]. The b-type fragment ions at *m/z* 278 (b2), *m/z* 406 (b3), *m/z* 503 (b4), and *m/z* 617 (b5), and the y-type fragment ions at *m/z* 115 (y1), *m/z* 340 (y2), *m/z* 427 (y3), *m/z* 541 (y4), *m/z* 638 (y5), and *m/z* 766 (y6) were all detected in the spectrum (Fig. 3A).

In addition to the b-type and y-type ions detected, some internal fragmentation ions and some ions that lost NH_3_ or H_2_O, or lost both, were also observed in the CID spectrum of the iturin precursor (Fig. 3A). The internal fragmentation ions at *m/z* 541.2 (NSXN)^+^, *m/z* 409 (SXN or NSX lacking one H_2_O)^+^, *m/z* 392 (NYN)^+^, *m/z* 340 (XN)^+^, *m/z* 323 (XN lacking one NH_3_)^+^, *m/z* 313.2 (SX)^+^, *m/z* 278 (NY or YN)^+^, and *m/z* 261 (NY or YN lacking one NH_3_)^+^ were detected in the CID spectrum. Other major ions were also detected, including *m/z* 1026.5 (M+H-NH_3_)^+^, *m/z* 1009.5 (M+H-2NH_3_)^+^, *m/z* 991.4 (M+H-H_2_O-2NH_3_)^+^, and *m/z* 975.5 (M+H-4NH_3_)^+^. Taken together, these results confirmed that the substance with molecular weight at *m/z* 1043.35 was iturin A.

**Fig 4:**
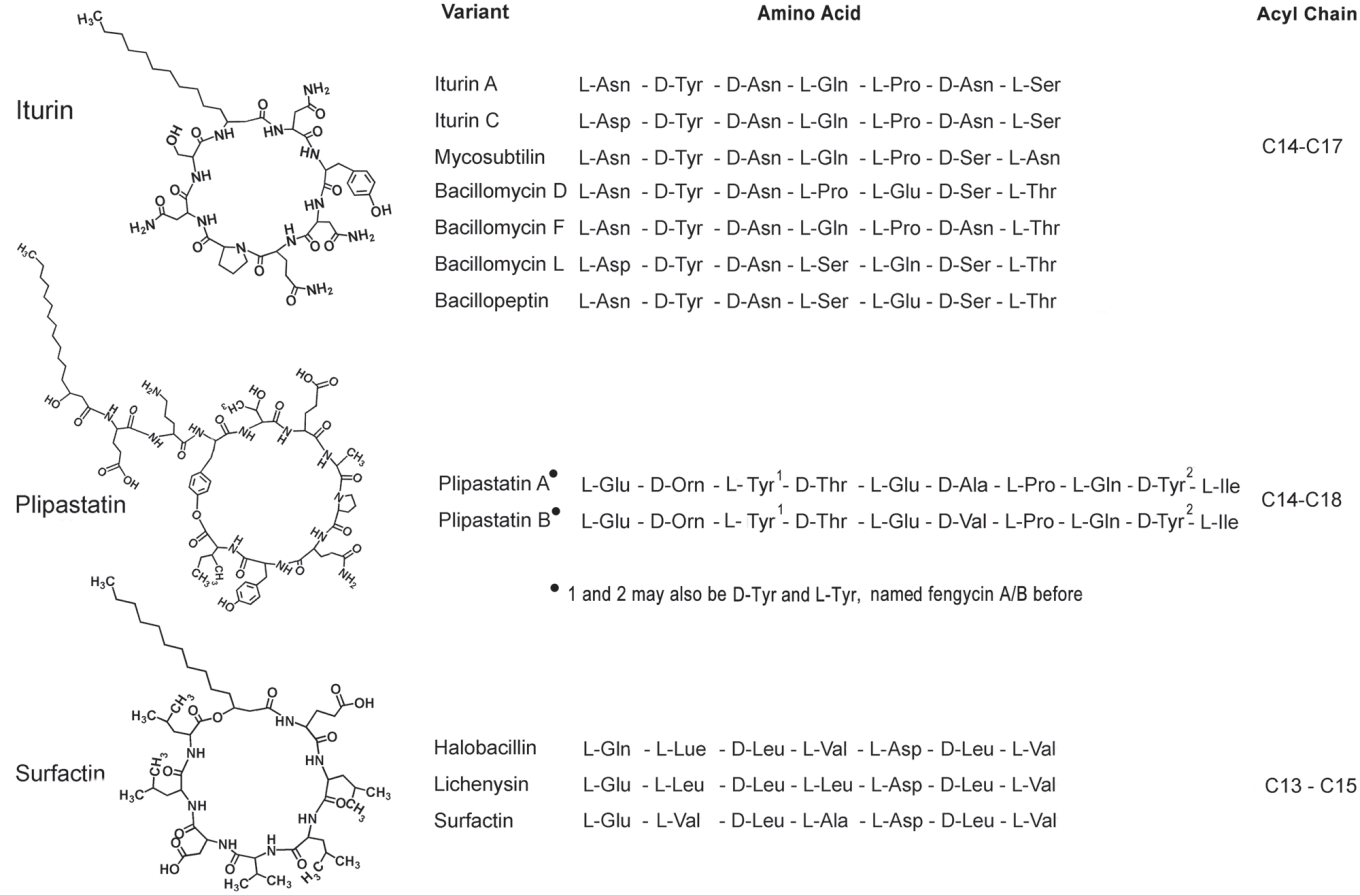
Primary structures of representative members within the three lipopeptides families produced by *Bacillus* spp. Plipastatin A/B with L-Tyr4 and D-Tyr10 was K^+^salt, with D-Tyr4 and L-Tyr10 was free form or TFA salt [16,26,27].

As for plipastatin (*m/z* 1463.90, precursor ion), two product ions at *m/z* 966 and *m/z* 1080 were detected (Fig. 3C). These are two typical ions for plipastatin A broken at the Glu-Orn and Orn-Tyr bonds (Fig. 3D, top panel) [42]. Furthermore, fragment ions at *m/z* 102.2 (Glu)^+^, 136.3 (Tyr)^+^, and internal fragmentation ions at *m/z* 226.05 (PQ)^+^, *m/z* 297.4 (APQ)^+^, *m/z* 302.20 (TQA)^+^, *m/z* 389. 4 (PQY)^+^, and *m/z* 1446.8 (M+H-NH_3_)^+^ were also detected in the CID spectrum (Fig. 3C). These results demonstrated that the substance with a molecular ion at *m/z* 1463.90 was plipastatin A. As the purification of plipastatin was conducted with H_2_O-CH_3_CN at the presence of trifluoroacetic acid (TFA) that could lead to the formation of free form or TFA salts, the plipastatin A should contain D-Tyr4 and L-Tyr10 (Figs. 3 and 4) based on a recent report [27]. Other precursor ions at *m/z* 1436, *m/z* 1450 and *m/z* 1478 were additionally identified as plipastatin A (Table 1). Precursor ions at *m/z* 1492 (M+H)^+^ and *m/z* 1506 (M+H)^+^, with product ions at *m/z* 994 and *m/z* 1108, belonging to plipastatin B, were also detected (Fig. 3D, bottom panel; Table 1) from strain S76–3. Iturin A (*m/z* 1043.35) and plipastatin A (*m/z* 1463.90) at free form or TFA salts were used for further analyses against FG.

**Table 1:** Identification of lipopeptides extracted from strain S76–3 through RP-HPLC and ESI-CID-MS.

Compounds	Mass peaks (*m/z*)	Assignments	References
Iturin A	1043, 1065, 1081 [M+H, Na, K]+ 1057, 1079, 1094 [M+H, Na, K]+	Asn-1 C14 iturinA Asn-1 C15 iturinA	[16,27,40,42]
Plipastatin	1436 [M+H]+ 1450 [M+H]+ 1464, 1486, 1502 [M+H, Na, K]+ 1478, 1500, 1516 [M+H, Na, K]+ 1492 [M+H]+ 1506 [M+H]+	Ala-6 C14 plipastatin A Ala-6 C15 plipastatin A Ala-6 C16 plipastatin A Ala-6 C17 plipastatin A Val-6 C16 plipastatin B Val-6 C17 plipastatin B	
Surfactin	1008, 1030, 1046 [M+H, Na, K]+ 1022, 1044, 1060 [M+H, Na, K]+ 1036, 1058, 1074 [M+H, Na, K]+ 1050, 1072, 1088 [M+H, Na, K] +	Leu-7 C13 surfactin Leu-7 C14 surfactin Leu-7 C15 surfactin Leu-7 C16 surfactin	

### MIC and microscope analysis of iturin A and plipastatin A against FG

To determine the MICs of iturin A and plipastatin A against conidial germination of FG, the two compounds were assayed at a range of concentrations. The two compounds exhibited different MICs against FG conidia. Iturin A displayed significant inhibitory activity at a concentration as low as 5 µg/ml, and complete inhibition of conidial germination at 50 µg/ml. For plipastatin A, although a similar partial inhibitory activity was seen at low concentrations, 100 µg/ml was required for complete inhibition (Table 2). No conidia germinated at the MIC, even after incubation on PDA for an additional 3 days at 28°C in S3 Fig., indicating that iturin A and plipastatin A at MIC had fungicidal activity and killed all of the conidial spores.

**Table 2:** Conidia germination inhibition tests of iturin A and plipastatin A against FG in half-strength YPG medium 12 hpi.

Concentration (µg/ml)	Inhibitory rate * (%)	
	Iturin A (*m/z* 1043.35)	Plipastatin A (*m/z* 1463.90)
0 (CK)	0.0 ± 0.0	0.0 ± 0.0
5	6.47 d ± 2.38	11.57 d ± 2.91
10	24.88 c ± 2.77	17.09 d ± 2.21
25	35.22 b ± 2.47	33.17 c ± 3.12
50	97.51 a ± 0.77	47.74 b ± 3.01
100	100 a ± 0.00	96.99 a ± 1.24
250	100 a ± 0.00	100 a ± 0.00

* Data presented are the average of three independent experiments ± standard error, and the same lowercase letters indicated no significant difference (P < 0.05).

**Fig 5:**
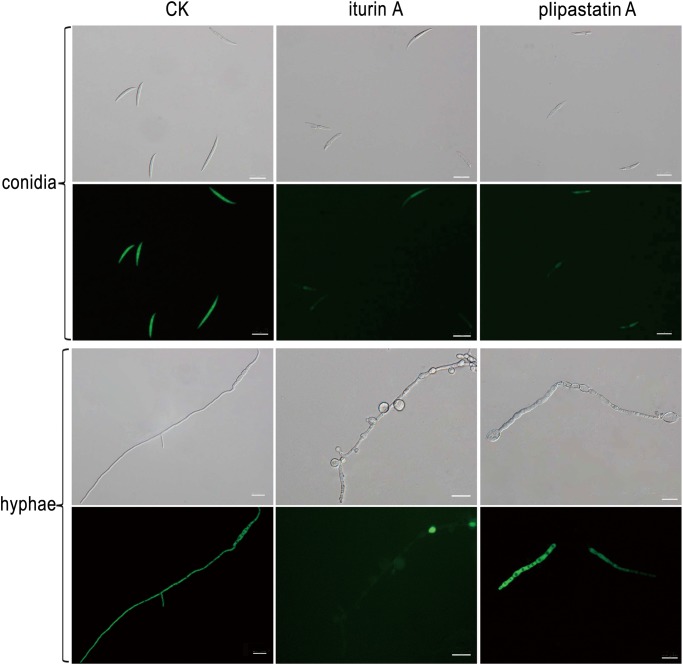
Optical and fluorescence microscopy analyses of FG treated with and without iturin A and plipastatin A. In control treatment (CK), conidia showed typical canoe shape and widespread fluorescence; hyphae had equal widths, even surfaces, and symmetrically distributed branching, with strong fluorescence along hyphae. In iturin A or plipastatin A treatments (iturin A, plipastatin A), conidia showed substantially deformed and damaged morphology, lateral expansion, and very weak or absent distribution of fluorescence. Iturin A caused substantial condensation and massive conglobation along hyphae with uneven surfaces, expanded widths, restricted branching, and very faint fluorescence in most regions. Plipastatin A caused severely distorted and condensed hyphae with increased vacuole sizes and deformed and conglobated apical tips, with interrupted fluorescence along hyphae. Bar = 25 µm.

To provide visual evidence of the action of iturin A and plipastatin A on the morphology and cellular contents of FG, conidial spores and hyphae treated with the two compounds were visualized by both optical and fluorescent microscopy. As shown in Fig. 5, in controls without the antagonistic compounds, conidial spores showed the typical canoe shape and hyphae grew actively, displaying equal widths and even surfaces with active branching. After staining with FDA, strong fluorescence was equally and fully distributed along all fungal structures, indicating the presence of active enzymes inside living cells and the integrity of the fungal membranes [25]. In contrast, treatment with iturin A or plipastatin A caused substantially deformed and damaged morphology for conidia: these showed lateral expansion and budding, and uneven surfaces. FDA staining of FG following iturin A or plipastatin A treatment revealed an interrupted, very weak, or absent distribution of fluorescence. Treatment of hyphae with iturin A caused substantial condensation and massive conglobation along hyphae, expanded widths and restricted branching, with very faint or absent fluorescence in most regions, but dense fluorescence signals at a few regions. Treatment with plipastatin A caused a similar pattern of damage for conidia, with very faint fluorescence. Hyphae treated with plipastatin A displayed substantial abnormalities: severely distorted and condensed structures with increased vacuole sizes and conglobated apical tips. The larger vacuoles apparently compressed cellular contents into a small space within cells and thus resulted in interrupted fluorescence along hyphae, with strong fluorescent signals in some regions but a lack of signal in most regions. FDA is a kind of enzyme activity probe to determine the cell viability through the membrane integrity. The weak fluorescent signals of FG treated with iturin A or plipastatin A indicated that their membrane may be severely damaged, especially in the iturin A treatments (Fig. 5). Therefore, iturin A and plipastatin A caused enormous structural and compositional changes to cell surfaces, cellular contents, cell membrane integrity and viability of fungal conidia and hyphae. Given the varied damage observed in FG following treatment with the two different compounds, we surmise that these two compounds may have different antagonistic modes of action against FG.

**Fig 6:**
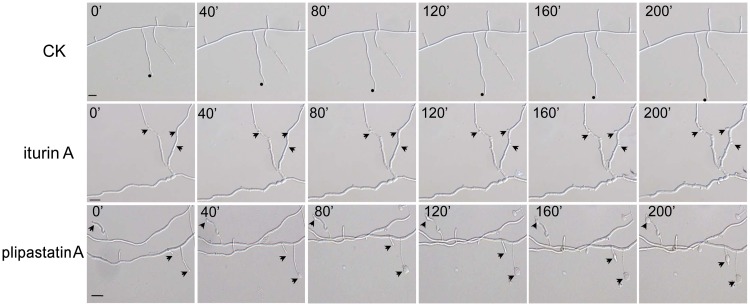
Dynamic processes of hyphae of FG as affected by iturin A or plipastatin A. Hyphae in control treatment (CK) had active apical growth with equal widths and many quickly elongating branches (circle); iturin A at MIC caused distortion and conglobation along hyphae, with lateral expansion and very restricted apical growth (iturin A, arrow); plipastatin A at MIC caused formation of conglobated structures, especially in young hypha and branch tips, resulting in either branch conglobation or restriction of apical growth (plipastatin A, arrow). The processes last 200 min. Bar = 25 µm.

To gain further insight into the dynamic processes involved in the interaction between iturin A or plipastatin A and FG, we used time-lapse imaging to monitor the antagonistic action of the two compounds during inhibition of hyphal development. As the two compounds kill conidia, hyphae from conidial spores geminated for 6 hours were used as the starting materials for the time-lapse imaging analyses. Pilot experiments revealed that visual indication of the antagonistic effects of the two compounds on FG hyphae were not clearly visible until 6 hours post treatment (data not shown). Hyphae that had been treated with iturin A or plipastatin A for 6 hours were therefore used for the time-lapse imaging. The time-lapse data was collected over a period of 200 min. The images are illustrated in Fig. 6. Under normal culture conditions lacking antagonistic compounds, FG hyphae developed actively, with filamentous structures of equal width with many branches, and had active hyphal apical growth with quickly elongating branches. Up to a 65 µm (n = 20) increase in branch length was observed in control hyphae (Fig. 6 and S1 Video). In contrast, iturin A and plipastatin A strongly inhibited and severely damaged hyphal growth, with obviously varied modes of action. Iturin A caused distortion and conglobation (more clearly visible at 200 min) along hyphae, with expanded width and very restricted apical growth; there was only a 5 µm (n = 20) increase in branch length observed (Fig. 6 and S2 Video). Plipastatin A caused formation of conglobated structures, especially in young hyphal and branch tips, resulting in either branch conglobation or restriction of apical growth, with only an 8 µm (n = 20) increase in branch length (Fig. 6 and S3 Video). Thus, iturin A mainly inhibited branch formation, while plipastatin A caused conglobation at hyphal tips. Both compounds caused lateral expansion of hyphae and restricted apical growth.

### Ultrastructures of FG conidia and hyphae treated by iturin A and plipastatin A

**Fig 7:**
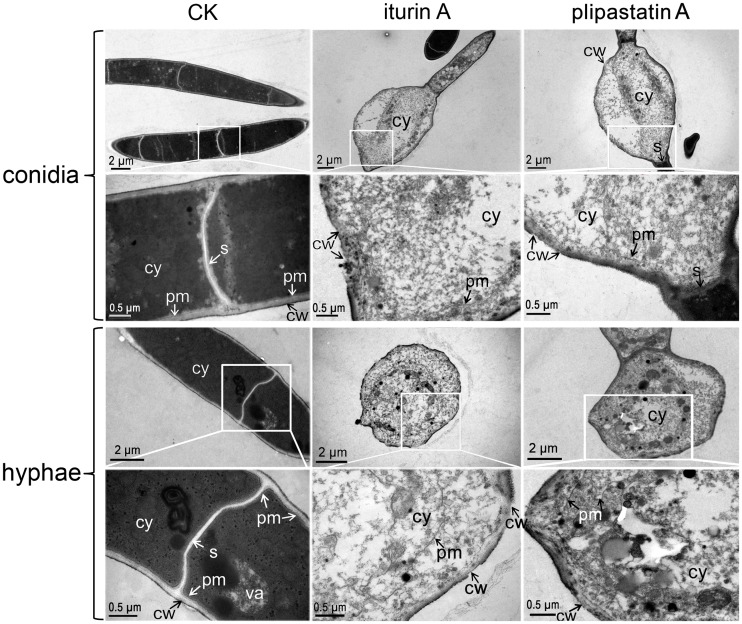
TEM analysis of FG conidia and hyphae treated with iturin A or plipastatin A. Conidia and hyphae lacking lipopeptide treatment (CK) showed dense cytoplasm with typical vacuoles, regular septa, and intact plasma membranes attached to electron-dense cell walls; Iturin A and plipastatin A caused complete structural destruction of conidia and hyphae, disorganized and sparse cytoplasm, uneven widths and gapped cell walls (cw with arrows) with indiscernible layers, dissociated cell organelles, and discontinuous plasma membranes. cy cytoplasm; va, vacuole; cw, cell wall; pm, plasma membrane; s, septum.

TEM analyses further illustrated the ultrastructural alterations of conidia and hyphae that resulted from treatment with iturin A or plipastatin A. As shown in Fig. 7, control FG conidial spores and hyphae produced regular cell walls, with equal widths and distinct layers, as well as septa with uniform composition and structure that spanned the entire width of the conidia and hyphae. Additionally, the control fungal cells had dense cytoplasm and had vacuoles with low (er) density contents. In contrast, treatment with iturin A or plipastatin A caused substantial structural destruction of conidia and hyphae, especially in cell walls and plasma membranes; treated cell walls had no discernible layers and uneven widths with thin or gapped structures. Furthermore, plasma membranes were detached from cell walls and septa, and became fragmented and distributed over entire cells. Septa were also severely damaged and had no distinct layers or structures. The damaged cell walls and membranes allowed the cell contents to leak out, resulting in less electronic density in the treated FG cells as compared to the control cells. Thus, iturin A and plipastatin A caused severe damage to the cell walls and plasma membranes of FG.

## Discussion

In modern pest management strategies, biological control agents have attracted a lot of attention as complementary or alternative methods to the use of conventional chemical fungicides. The dissection of the antagonistic mechanisms of active compounds derived from the biocontrol organisms is vitally important for the efficient application and proper deployment of such compounds in agriculture.

ESI-CID-MS analyses revealed that the cyclic peptide of iturin A (*m/z* 1043.35) was broken to generate two main linear acylium ions, Pro-Asn-Ser-βAA-Asn-Tyr-Asn-Gln-Co^+^ and Tyr-Asn-Gln-Pro-Asn-Ser-βAA-Asn-CO^+^ (Fig. 3A and 3B). A series of b-type and y-type ion fragments derived from dissociations were detected. These product ions congruently indicated that the most abundant fraction of the iturin class of lipopeptides, at *m/z* 1043.35, was iturin A. For the identification of plipastatin, fragment ions of *m/z* 966, 1080 and *m/z* 994, 1108 (Fig. 3C and 3D) can be considered as ‘fingerprints’ in discrimination of plipastatin A and plipastatin B, respectively [42]. Fragment ions at *m/z* 966 and 1080 can be explained as the neutral loses of [fatty acid-Glu]^+^ and [fatty acid-Glu-Orn] ^+^ from the N-terminal segment of plipastatin A with Ala at position 6 (Fig. 3D, top panel). Hence, the most abundant compound of the plipastatin class of lipopeptides (*m/z* 1463.90) with product ions at *m/z* 966 and 1080 was identified as plipastatin A.

MIC tests showed that iturin A at 50 µg/ml and plipastatin A at 100 µg/ml completely inhibited FG conidium germination; no conidia germinated after further incubation on PDA for an additional 3 d. In contrast, surfactin purified from strain S76–3 did not show antifungal activity (Fig. 2). This is different from one previous report that surfactin from *B*. *licheniformis* BC98 was responsible for the antifungal activity against *Magnaporthe grisea* [43]. Our results indicated that both iturin A and plipastatin A have fungicidal activity, that both are able to kill conidia, and that iturin A is active at lower concentrations than plipastatin A. The difference between iturin A and plipastatin A may result from their structural properties: the two compounds contain different amino acids in the peptide cycle, and, further, plipastatin A contains two more amino acids in its longer fatty acid tail (n = 16) compared with that in iturin A (n = 14) (Fig. 4). The lengths of fatty acid tails and amino acid sequences of peptide cycles have been shown to have a vitally important impacts on the antifungal activities of the lipopeptides [19,22,46]. The properties from iturin A may favor for its affinity and/or interaction with the fungal membranes from FG.

Optical microscopy showed that treatment with iturin A and plipastatin A caused many abnormalities in conidia and hyphae, and that these structures had either faint or absent fluorescence, or unevenly distributed fluorescence signals after FDA staining. FDA is a kind of enzyme activity probe that is recognized by nonspecific esterases, and this recognition releases fluorescence once it enters living cells, thus serving as an indicator of intracellular enzymatic activity [25]. These results indicated that the cellular contents in FG may become inactive after treatment with the antagonistic lipopeptides, and that this inactivation was not reversible. Whether or not the lipopeptides directly or indirectly inhibit the catalytic activities of the cellular enzymes remains an open question. Furthermore, TEM analysis showed that treatment with the two compounds caused severe alterations to the overall ultrastructures of hyphal cytoplasms, cell walls and membranes of conidia. Thus, iturin A and plipastatin A caused damage to key fungal structures.

Plipastatin A caused vacuolation along hyphae (Fig. 5) and conglobation on young hyphae and branch tips (Fig. 6 and S3 Video). These results suggest that the plipastatin A may disturb cellular composition and organization to increase vacuole sizes and in turn to depress cellular contents into smaller spaces (Fig. 5). Hyphae of *Rhizopus stolonifer* treated by plipastatin (no assignment of the plipastatin) from one *B*. *subtilis* fmbJ showed uneven cell wall and large vacuole inside the cells [44]. Plipastatin A was considered as the inhibitory compound from *B*. *subtilis* IB against *F*. *graminearum* and no antagonistic action mode has been shown [45]. Zhao et al. [25] showed that plipastatin A had no obvious effect on the morphology or cellular enzymes of *F*. *oxysporum* as evaluated by FDA staining and microscopy. These inconsistent results for plipastatin A suggest that the unique fungal cell structures and compositions may have a decisive role in the interaction with plipastatin A, although the fungus used in this study and species from Zhao et al. [25] belong to the same genus (*Fusarium*). The composition of phospholipids and the amount of sterols in the fungal membranes have been proposed to be important for their sensitivity to plipastatin [26]. A very recent study has reported that high contents of fungal ergosterol positively correlated with increased plipastatin tolerance in fungal pathogens that infect plants due to the buffer fluidity changes in the fungal membranes [46]; a sensitivity to plipastatin was often accompanied with the decreased fluidity buffering capacity, lower ergosterol content and shorter phospholipid fatty acyl chains in fungal membranes. Various plant fungal pathogens may have different compositions of phospholipids and ergosterols, and thus display different sensitivities to plipastatin [46]. Monitoring the (hypothetical) dynamic processes of antagonistic actions of individual plipastatin during such assays may be an important experimental approach for dissecting their modes of antifungal action.

Transmission electron microscopy revealed widely gapped cell walls and severely damaged plasma membranes of the conidia and hyphae after treatments with iturin A and plipastatin A (Fig. 7). To our knowledge, this is the first evidence that iturin and plipastatin can cause gapped cell walls in filamentous fungi. The fungal cell wall protects the cell from changes in osmotic pressure and other environmental stresses and is considered the “carbohydrate armour” of the fungal cell [47]. The fungal plasma membrane is responsible for maintaining cell order and integrity, and its integrity is imperative to the survival of a fungus [48]. We show that iturin A and plipastatin A cause damage to these structures, and we conclude that such damage is the basis of the severely inhibited hyphal growth and the failure of FG conidia to germinate observed in the lipopeptide treated samples.

Strain S76–3 was from wheat spikes of the FHB-susceptible cultivar Annong 8455 grown in a region with frequent FHB epidemics. Annong 8455 has been widely used as an FHB-susceptible control in FHB resistance assays [38]. Strain S76–3 may have unique characteristics for survival and adaptability in agro-ecosystems with high disease pressure; it may thus be superior as a biocontrol agent in wheat fields when compared with the performance of other antagonists isolated from non-*Fusarium* pathogen infected environments. Iturin has been shown to be active against FG in field test [20]. Two most abundant lipopeptides produced by this strain, iturin and plipastatin, displayed different antagonistic mechanisms against FG. In addition, surfactin, also produced by strain S76–3 (Fig. 2), has been shown to have synergistic effects both to iturin on haemolytic activity [49] and to plipastatin on bacterial inhibition [50]. Therefore, we envisage that the strain S76–3 capable of producing three kinds of lipopeptides is a promising biocontrol agent for use in the effective and environmental-friendly control of FG-associated cereal diseases and mycotoxins in agriculture.

## Supporting Information

S1 Fig Neighbor-joining phylogenetic tree of strain S76–3 and other homologous *Bacillus* spp. based on 16S rDNA sequences. Values for frequencies less than 50% are not given. The scale bars represent the number of substitutions per base position.
(TIF)

S2 Fig Comparsion of lipopeptide produced by S76–3 and commercial iturin A and surfactin homologues through RP-HPLC. Iturin and surfactin produced by strain S76–3 showed the same retention times (iturin, 22.755–27.586 min; surfactin, 51.704–54.893 min) as the respective standards purchased from Sigma-Aldrich did at UV spectrum of 214 nm.
(TIF)

S3 Fig Germination tests of FG conidia at the presence of iturin A or plipastatin A at MIC 12 hpi. Conidia treated with iturin A and plipastatin A at MIC for 12 h were centrifuged, resuspended in water, spread on PDA plates, and cultured for 3 d at 28°C.
(TIF)

S1 Table Physiological and biochemical analysis of strain S76–3. (DOCX)

S1 Video Dynamic process of hyphal growth of FG under normal culture conditions, without antagonistic compounds. FG hyphae actively developed with equal widths and symmetrical distributed branches, and had active hyphal apical growth with quickly elongated branches. The process documented in this video lasts 200 min.
(MPG)

S2 Video Dynamic process of hyphae growth of FG with the presence of iturin A. Iturin A caused distortion and conglobation along hyphae with expanded widths and restricted apical growth and branch length.
(MPG)

S3 Video Dynamic process of hyphae growth of FG with the presence of plipastatin A. Plipastatin A caused the formation of conglobated structures, especially in young hyphae and branch tips, resulting in either branch conglobation or restriction of apical growth and branch length.
(MPG)
