# Supplementary figures and images for: Antagonistic Mechanism of Iturin A and Plipastatin A from Bacillus amyloliquefaciens S76-3 from Wheat Spikes against Fusarium graminearum
Source: PLoS One. 2015 Feb 17;10(2):e0116871. doi: 10.1371/journal.pone.0116871 (PMC4331432; doi:10.1371/journal.pone.0116871)

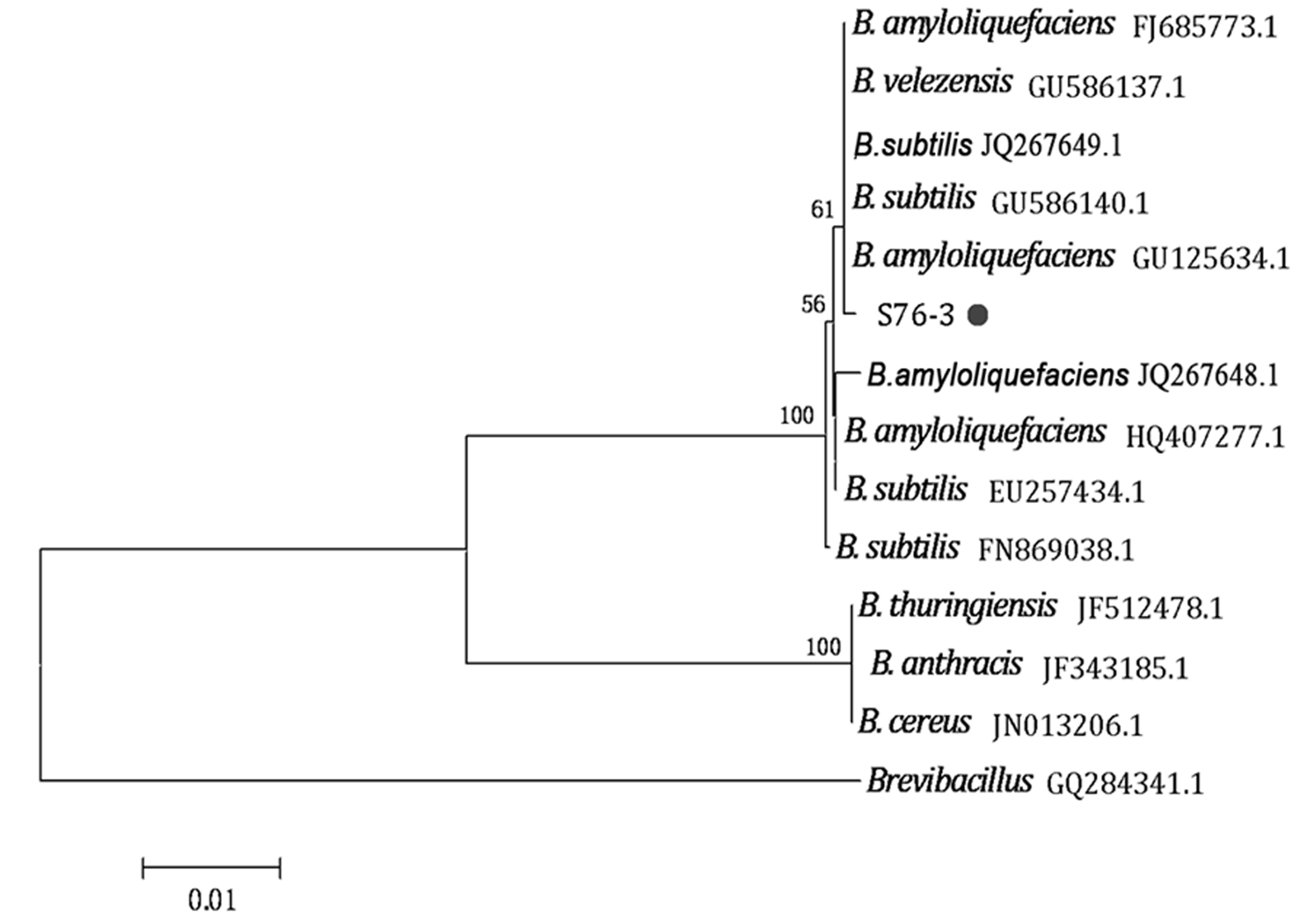

Supplement: S1 Fig — Values for frequencies less than 50% are not given. The scale bars represent the number of substitutions per base position. (TIF) [file pone.0116871.s001.tif]

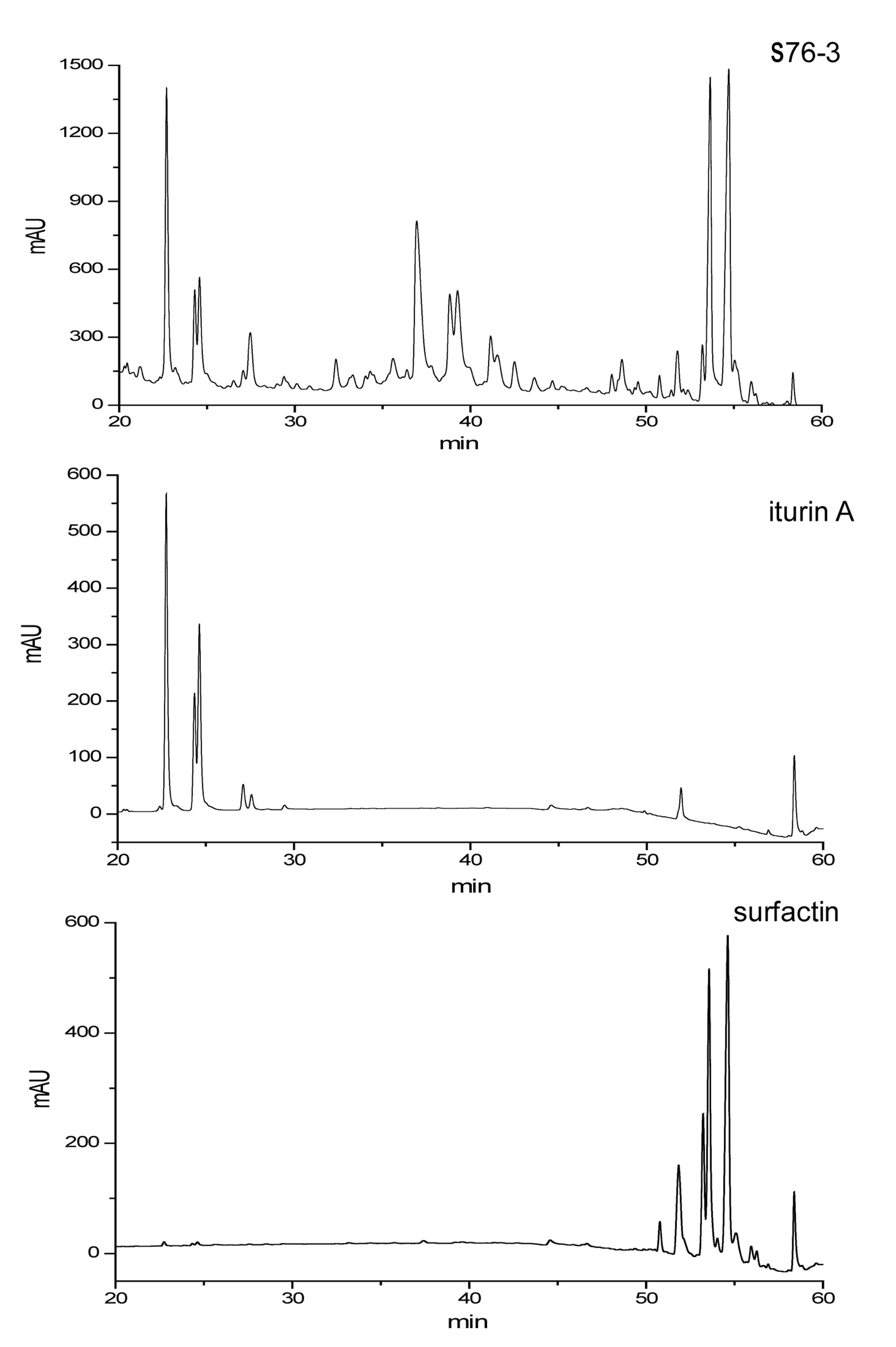

Supplement: S2 Fig — Iturin and surfactin produced by strain S76–3 showed the same retention times (iturin, 22.755–27.586 min; surfactin, 51.704–54.893 min) as the respective standards purchased from Sigma-Aldrich did at UV spectrum of 214 nm. (TIF) [file pone.0116871.s002.tif]

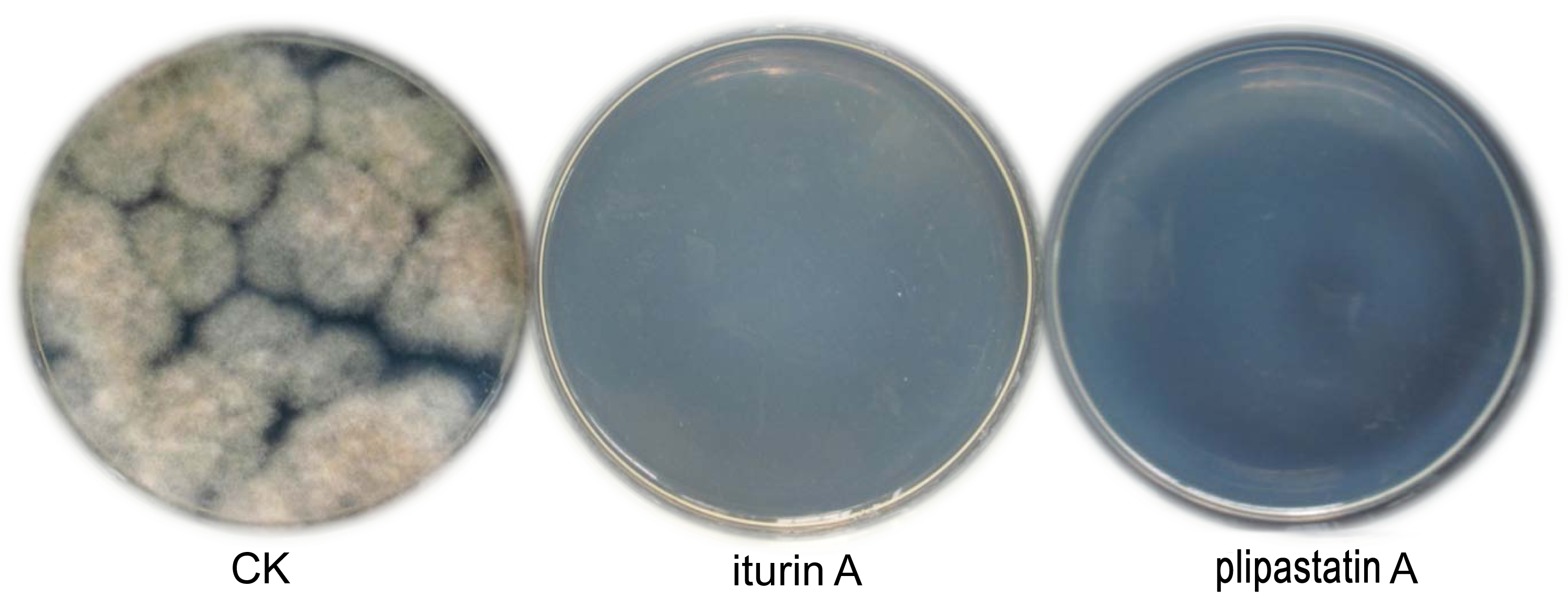

Supplement: S3 Fig — Conidia treated with iturin A and plipastatin A at MIC for 12 h were centrifuged, resuspended in water, spread on PDA plates, and cultured for 3 d at 28°C. (TIF) [file pone.0116871.s003.tif]
