# Supplementary material for: Antagonistic Mechanism of Iturin A and Plipastatin A from Bacillus amyloliquefaciens S76-3 from Wheat Spikes against Fusarium graminearum
Source: PLoS One. 2015 Feb 17;10(2):e0116871. doi: 10.1371/journal.pone.0116871 (PMC4331432; doi:10.1371/journal.pone.0116871)
Supplement: S1 Table — (DOCX) [file pone.0116871.s004.docx]

**Table S1.** Physiological and biochemical analyses of strain S76-3.

| Culture condition | S76-3 | *B. amyloliquefaciens* ^a^ (52 strains) | *B. subtilis* ^a^ (131 strains) |
| --- | --- | --- | --- |
| Carbon utilization |  |  |  |
| D-Fructose | + | 100 | 100 |
| Sucrose | + | 100 | 100 |
| D-Glucose | + | 100 | 100 |
| Maltose | + | 98 | 100 |
| Lactose* | + | 84 | 49 |
| D-Xylose | + | 71 | 89 |
| Rhamnose* | - | 0 | 2 |
| L-Arabinose | + | 82 | 98 |
| D-Mannose | + | 63 | 94 |
| D-Sorbitol | + | 88 | 88 |
| Gelatin liquefaction | + | + | + |
| Hydrolysis of starch | + | + | + |
| Peptonization of milk  (10% skim milk) | + | + | + |
| Hydrolysis of Tween 80 | + | + | NR |
| Tolerance to 7% NaCl | **+** | **+** | **+** |
| Tolerance to 10% NaCl* | **+** | + | **-** |
| Citrate utilization | + | + | + |
| different colors on potato piece * | Brown | Brown | Pink |
| Growth at 51°C * | - | **-** | **+** |

^a^ The numbers in the table represent the percentage of the strains that showed positive reactions in the 52 *Bacillus amyloliquefaciens* or the 131 *Bacillus subtilis* strains. The characteristics of *B. amyloliquefaciens* that are different from *B. subtilis* are marked by asterisks (*).

MS: most strains had positive reactions.

NR: no reference.

+: positive reaction.

- : negative reaction.
